# Gentle Africanized bees on an oceanic island

**DOI:** 10.1111/j.1752-4571.2012.00252.x

**Published:** 2012-11

**Authors:** Bert Rivera-Marchand, Devrim Oskay, Tugrul Giray

**Affiliations:** 1 Department of Science and Mathematics, Inter American University of Puerto RicoBayamón, PR, USA; 2 Department of Biology, University of Puerto RicoSan Juan, PR, USA; 3 Department of Agricultural Biotechnology, Namik Kemal UniversityTekirdag, Turkey

**Keywords:** Africanized honeybee, *Apis mellifera*, defense, *Varroa*

## Abstract

Oceanic islands have reduced resources and natural enemies and potentially affect life history traits of arriving organisms. Among the most spectacular invasions in the Western hemisphere is that of the Africanized honeybee. We hypothesized that in the oceanic island Puerto Rico, Africanized bees will exhibit differences from the mainland population such as for defensiveness and other linked traits. We evaluated the extent of Africanization through three typical Africanized traits: wing size, defensive behavior, and resistance to *Varroa destructor* mites. All sampled colonies were Africanized by maternal descent, with over 65% presence of European alleles at the S-3 nuclear locus. In two assays evaluating defense, Puerto Rican bees showed low defensiveness similar to European bees. In morphology and resistance to mites, Africanized bees from Puerto Rico are similar to other Africanized bees. In behavioral assays on mechanisms of resistance to *Varroa*, we directly observed that Puerto Rican Africanized bees groomed-off and bit the mites as been observed in other studies. In no other location, Africanized bees have reduced defensiveness while retaining typical traits such as wing size and mite resistance. This mosaic of traits that has resulted during the invasion of an oceanic island has implications for behavior, evolution, and agriculture.

## Introduction

Introduced invasive species are considered a problem throughout the world, impacting biodiversity, economy, and human well-being (Pimentel et al. 2000). However, invasive species also are considered models for the study of evolutionary processes, such as natural selection and genetic drift because they may face environments different from their place of origin (Reznick and Ghalambor 2001; Lee 2002). These environmental differences will be more notable if the organism arrives from a continent to an isolated island. This is because in comparison with continents, islands tend to have more limited resources, space, and predators, all of which may affect the arriving species (McNab 1994). The presence of Africanized bees on the Caribbean island of Puerto Rico offers a unique opportunity to study this well-known invasive species from the mainland on an oceanic island and to evaluate any genetic or behavioral differences (see Rivera-Marchand 2006; Rivera-Marchand et al. 2008).

Africanized bees arrived to Puerto Rico in 1994 (Cox 1994), the first report of these bees on an oceanic island. The Africanized honeybee is considered to be an invasive species because of its infamous negative effect on human health and their effective competition for resources with native organisms such as for nesting locations (Schneider et al. 2004). Considered a hybrid between the African race (*Apis mellifera scutellata*) and several European races, Africanized bees in continental America have retained tropical adaptations including high defensive behavior (Breed et al. 2004), mite resistance, and smaller size throughout their range (Schneider et al. 2004) although they do show considerable genetic and morphological differences from the original subspecies and invading populations (Francoy et al. 2009). We examined the extent of Africanization of the feral honeybees in Puerto Rico using one nuclear and one mitochondrial molecular marker (Nielsen et al. 2000; Suazo and Hall 2002) and evaluated three typical traits of these bees: wing size, defensive behavior, and resistance to *Varroa* mites (Breed et al. 2004; Schneider et al. 2004).

We hypothesized that in the island environment defensive behavior of Africanized bees would be reduced both because of selective forces facing colonists on islands, such as lack of natural enemies and limited resources and because of a specific trade-off between defense and foraging described in honeybees (Giray et al. 2000; Rivera-Marchand et al. 2008). In preliminary observations, the Africanized bees of Puerto Rico seemed to be less defensive than expected while retaining their typical size and mite resistance. Moreover, a dramatic decrease in reported attacks since the arrival of Africanized bees in Puerto Rico (Rivera-Marchand 2006) supports the hypothesis of decreased defensiveness. For instance, there had been four deaths in the first 4 years of the arrival of Africanized bees and none in the last 10 years. This pattern is significant in a simple runs test. The number of incidents where bees attacked people, as investigated in newspaper archives, shows a 100-fold decrease in numbers in the same time period. These data may indicate reduced defensiveness in Africanized bees from Puerto Rico, although public awareness could account for part of the decrease in incidents (Rivera-Marchand 2006).

In addition, we expected a potential change in apparently unrelated characteristics such as morphology and ectoparasite resistance mechanisms together with defense. Groups of linked traits such as these, called behavioral syndromes, have been observed in honeybees (reviewed by Hunt 2007; see also Giray et al. 2000; Tsuruda and Page 2009; Giray et al. 1999). The prevalence of the ectoparasitic mite *Varroa* which arrived to the island in 1984 (Martin et al. 1998) may have contributed to the disappearance of feral European bees (De Guzman et al. 1999) and the success of Africanized bees as suggested in other places (Martin and Medina 2004). Alternately, the defensive behavior, ectoparasite resistance, and morphology could be evolving independently. Then, these bees would have reduced defensiveness and still retain their resistance to *Varroa destructor* mites, typical of Africanized bees (Martin and Medina 2004; Mondragón et al. 2005), and their smaller size, thought to be a tropical adaptation (Harrison and Hall 1993).

## Materials and methods

### Study site and colonies

We performed the study on the Caribbean island of Puerto Rico including the adjacent islands of Vieques and Culebra from 2001 to 2006. Puerto Rico is a 9104-km^2^ island with an irregular topography, fairly stable temperature (maximum = 30°C; minimum = 24.8°C) but irregular rainfall. Maximum annual rainfall is reported in the northeast at ∼563 mm, and the minimum, recorded from the south, is ∼7 mm.

To determine behavioral characteristics of feral bees in Puerto Rico, we collected feral colonies, placed them in standard hive, and brought them to a standard colony population size (ca 20 000 workers). We transferred most of the combs, brood, and workers along with the queen to typical Langstroth hives in a common apiary. We tested defensive behavior and mite levels after colonies reached the standard size.

### Africanization

To evaluate Africanization in Puerto Rico, we collected worker bees from 112 feral hives during the period from 2002 to 2006 (Figure S1). To ensure colony origin, we only sampled pollen foragers returning to the hive or newly emerged bees inside the hive (Giray et al. 2000). The sampled colonies are representative of the six ecological life zones (according to the Holdridge classification) and of the maximum and minimum elevations of the island (Ewell and Whitmore 1973).

We placed sampled worker bees in 95% ethanol and kept them at −20°C until DNA was extracted. We performed DNA extractions with the Qiagen® DNA Easy Extraction Kit (Qiagen Inc., Valencia, CA), following the animal tissue protocol. To determine Africanization by maternal descent, we followed the RFLP protocol and regional classification of *A. mellifera* subspecies of Nielsen et al. (2000). The method distinguishes Africanized bees from Western European (*A. m. mellifera* and *A. m. iberensis*), Eastern European (*A. m.* *ligustica*, *A. m. carnica* and *A. m. caucasica*), and Egyptian (*A. m. lamarckii*) origin. In Puerto Rico, a law passed in 1996 prohibits entry of any bees other than queen bees from Hawai’i (which are Italian bees; Kona Apiaries). As a precaution, we screened 15 bees from different colonies for all mitotypes. Because the only reported honeybees on the island, other than Africanized, are of eastern European origin (e.g. *A. m. ligustica*) and the samples did not demonstrate the presence of other races, we screened the remaining samples with the method for identifying Eastern European bees versus Africanized bees. We amplified a region of the rDNA of approximately 964 bp. The PCR product was then digested with the restriction enzyme EcoRI. This enzyme digests the PCR product of the Eastern European bees into two bands of 480 and 484 bp, yet does not digest the PCR product of Africanized bees. We visualized the samples in a 1% agarose gel stained with ethidium bromide.

To determine the presence of European genes, we genotyped Africanized queens using a RFLP method developed by Suazo and Hall (2002). We sampled six drones from 16 hives of the previously determined Africanized colonies (Suazo and Hall 2002). Drones are produced from unfertilized eggs, and they represent the genotype of the queen. Moreover, by being haploid, the banding pattern is easier to interpret. All drones were obtained as pupae and stored until DNA was extracted using the same protocol as for worker bees. We amplified a region of the nDNA called s-3, and the PCR product was digested with AluI, a restriction enzyme that produces a particular banding pattern with four fragments in European bees and five specific fragments in African bees.

### Wing size

We measured the wings lengths of 244 bees of 28 colonies (8–9 bees/colony) following the FABIS method (Sylvester and Rinderer 1987). Africanized bees are identified by having smaller wings than European bees. We removed the wings at the hinge, placed on a slide, and projected an image of them on a flat surface with an opaque projector. A 1-cm scale was placed on the slide to determine the actual size of the wing.

We examined museum collections to determine whether Africanized bees replaced European bees after their arrival in 1994 (Cox 1994). Wing lengths of 223 honeybees present in two museum collections dating from three time periods (1900–1983; 1984–1993; 1994–2003) were determined using the FABIS method (Sylvester and Rinderer 1987) with the exception that instead of removing wings, the bees were placed within a foam wing spreading block and their wings were spread under a slide. We also compared numbers of honeybees and native bees belonging to 27 species in collections to determine whether collecting effort differed over time and could explain some of the changes in numbers of honeybee specimens.

### Defensive behavior

We evaluated defensive behavior with a repeated behavioral rank assay and a repeated sting assay (Giray et al. 2000; Guzmán-Novoa et al. 2003). The behavioral rank assay was performed twice, with 1 week between trials, and the scores for a colony were averaged. We kept 12 Africanized and 12 Italian colonies headed by queens imported from Hawai’i in a common apiary. Two puffs of smoke were given at the entrance and one puff on top after opening the colony. One frame was removed and behaviors of the bees were observed. Four different behaviors related to defense – running, flying, hanging from the frame, and stinging – were ranked from 1 to 4. To maintain consistency of the assay, the same person performed all evaluations blind to colony race. We added the scores for each behavior; the total for each race was averaged and compared with each other and with data from Africanized and European bees of Mexico (Giray et al. 2000).

The sting assay included two trials performed 1 week apart on nine feral Africanized colonies in the common apiary. A 6 × 5 cm black leather patch was waved in front of the colony with an added disturbance of physical impact to the box (Giray et al. 2000). The time to first sting and number of stings deposited on the leather patch in 60 s after the first sting were recorded; results from the two trials were averaged for analysis. Colonies that did not sting after 2 min of start of testing were given a score of 0 stings. The averaged total number of stings and time to first sting data were compared to data of European bees studied in Illinois (Giray et al. 2000).

### Mite resistance

Among the mechanisms that may maintain a low *Varroa destructor* mite infestation are hygienic behavior (Boecking and Spivak 1999) and grooming (Mondragón et al. 2005). We evaluated mite infestation at the brood and colony levels. *Varroa* mites can be found within capped brood cells, where most of their life cycle occurs, as on adult bees. Brood infestation was evaluated by removing 100 pupae per colony and inspecting for *Varroa destructor* mites (Mondragón et al. 2005).

We assessed colony-level infestation of nine previously untreated colonies as well as ten colonies treated with Apistan® 1 year prior to the experiment. For both samples, mite abundance was assessed by applying Apistan® (Guelph, Ontario, Canada) overnight and collecting fallen mites with a sticky trap placed on the bottom board of the hive box. To evaluate the possible differences between previously untreated and treated hives, we compared average mite fall in these two groups of colonies.

We also examined grooming behavior in the Africanized bees at the colony level by quantifying natural mite fall. We placed sticky traps on the bottom board of nine hives overnight. The sticky trap was screened from the bees, so they could not access the mites after they fell. We inspected fallen mites for damage which was inferred to be caused by the bees.

We evaluated hygienic behavior of nine Africanized bee colonies with a frozen brood assay (Spivak and Downey 1998). We removed one frame of capped brood from the colony. We placed a metal square within a section of the frame that included 100 cells, slowly poured liquid nitrogen (∼300 mL) into the section, waited until the frozen brood thawed, and returned the frame to its hive of origin. The frame was inspected daily for brood removal during a period of six days. A colony that removes 95% of the frozen brood within two days is considered hygienic (Spivak and Downey 1998).

We examined grooming behavior of Africanized and Italian bees with a comparative behavioral assay similar to a previously published method (Aumeier 2001). Africanized bees have been reported to groom and damage *Varroa* mites, while Italian bees are less efficient at mite removal and damage (Mondragón et al. 2005). With an insect sampling vacuum (Model 5911, Type 1, 12V DC; BioQuip, Rancho Dominguez, CA), we sampled 450 worker bees from four Italian colonies and 450 workers from four Africanized colonies (see Giray et al. 1999, 2000; Rivera-Marchand et al. 2008). The workers were first anesthetized with carbon dioxide to allow manipulation. We then placed ten worker bees representing each of the sampled colonies in separate covered plastic containers of 10 cm in diameter and 2.57 cm deep (SOLO Cup Company, Lake Forest, IL). Small holes were made on the cover to allow the passage of air. *Varroa* mites were collected with a small damp paint brush from colonies different to those used in the assay. After the bees had recovered from the carbon dioxide, one mite was placed in each container through one of the air holes. Bee behaviors were recorded with a video camera. The behaviors we scored were biting the mite, unsuccessful attempt to groom, and successful grooming.

To be able to compare the time spent grooming and intensity of grooming by Africanized and European bees, we had to examine only the unsuccessful grooming events. This is because European bees never exhibited successful grooming behavior. We used the video to determine this amount of time which began at the moment the mite attached itself to the bee and ended when the bee gave up grooming. The time to quit grooming for the Africanized and Italian bees was compared.

To determine whether grooming and biting are behaviors special to bees of a certain age or task group, we sampled nurses, guards, and foragers from three colonies. The trials were performed as in the previous assay except that the containers were covered with red paper. Because bees are less sensitive to red light (von Frisch 1967), covering the containers with red paper simulated conditions similar to the dark colony. Three trials that included ten workers of each task group per colony were placed in the container and filmed with a video camera. Data were recorded as in the previous trial.

### Statistical analyses

With the exception of the analysis of average effect size, data were analyzed using the JMP 5© software (SAS Institute Inc., Cary, NC 2002). We tested for normality and verified that all data met the assumptions of each statistical test. The relative representation (under and over representation) of honeybees in the museum data was analyzed with a likelihood ratio test. Ranked defensive behaviors were added, and comparisons between Africanized bees in this study and Africanized and European bees from Mexico (Giray et al. 2000) were performed with the nonparametric Wilcoxon signed-rank test. Stinging behavior of Africanized and European bees from a common apiary was analyzed by calculating the 2-day trial average of time to sting and number of stings on a leather patch and comparing them with *t*-tests.

In a meta-analysis of the differences in defensiveness of Africanized and European bees, we compared the average effect size (Cohen’s d, based on pooled standard deviations of the samples) from this study with others (see Table 1). We chose to compare the effect sizes because of different scales and assays used in each study and because the standardized effect size conveys the size of an effect relative to the variability in the different populations (Hartung et al. 2008). Because each study represents measurements on a particular population of Africanized bees and cannot be considered independent measurements, we compared averages for each study. The different tests and measures used in studies compared for effect size are indicated in Table 1. In different tests, a lower or a higher score may indicate greater defensiveness; therefore, we report absolute values for effect sizes.

**Table 1:** Effect size for defense test differences between Africanized (A) and European (E) bees

Test (measure)	Mean: A (SD); E (SD)	*d*	Reference (Ave. d)
Flag + impact (a)	56.6 (44)/57.7 (56.7)	0.02*	This study, E: 13
Flag + impact (b)	220 (142); 197 (201)	0.13*	This study, E: 13
Behav. assay (c)	6.25 (2.19); 5.05 (2.59)	0.48*	This study (*d* = 0.21)*
Flag (a)	109.5 (18.6); 19 (13.7)	5.55	51 (*d* = 5.55) **
Flag (b)	62.67 (30.6); 14.67 (25.4)	1.71	58
Flag (a)	1.53 (0.289); 22.07 (17.70)	1.64	52 (*d* = 1.68)
Box (d)	37.3 (16.66); 7.5 (8.82)	2.24	23
Pheromone (e)	126.8 (31.59); 35.7 (39.93)	2.53	23
Flag (b)	88.3 (34.54); 7.6 (15.92)	3.00	23 (*d* = 2.59)
Flag (b)	125 (17.32); 38 (12.12)	5.82	53 (*d* = 5.82)
Behav. assay (c)	11.5 (1.88); 5.25 (2.42)	2.88	13 (*d* = 2.88)
Flag (a)	11.7 (2.98); 51.3 (15.92)	3.46	36
Flag (a)	9.3 (1.71); 58.7 (6.48)	10.4	36
Flag (a)	8.3 (1.21); 52.0 (7.88)	7.75	36 (*d* = 7.21)
Pheromone (a)	11.16 (1.16); 16.74 (1.63)	3.94	54 (*d* = 3.94)
Glove (a)	3.3 (1.5); 15.9 (6.6)	2.63	61
Glove (b)	48.6 (57.7); 0.1 (0.2)	1.19	55 (*d* = 1.91)

* First column gives the test and specific measure used from the test. Different measures used in the studies are indicated by a letter in parentheses in the first column: (a) time to first sting (sec.); (b) number of stings in unit time; (c) rank sum of four indicator behaviors (see Methods); (d) percentage of defending bees that stung a flag; (e) recruitment of defending bees to the hive entrance. The second column gives the means for Africanized bees –A and European bees – E; the standard deviation for the means is given in parentheses (SD). The third column reports the effect size for the A versus E comparison for the particular test measure. The last column gives the reference number for the study, and if multiple tests were employed in the study in parentheses, an average effect size (Ave. d) is given. All three effect sizes from this study represent outliers in a Huber’s outlier test (Figure S2).

** Statistics calculated from plots.

We evaluated the possible differences between previously untreated and acaricide-treated hives by comparing their average mite fall with a *t*-test. Grooming behavior was evaluated by comparing time to quit grooming for the Africanized and Italian bees with a *t*-test. We performed an anova to compare grooming and biting behavior among age groups of Africanized bees.

## Results

### Africanization

**Figure 1:**
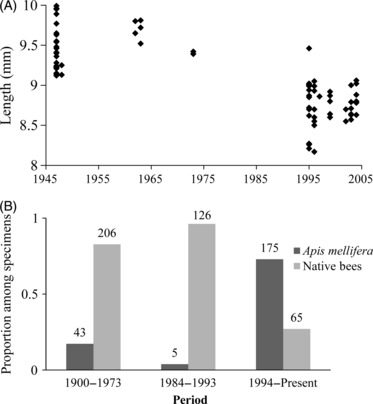
Honeybees in museum collections. Figure 1(A): Wing lengths of honeybees of Puerto Rico from 1947 to 2005. Bees with wing lengths <9 mm are considered Africanized, while those >9 mm are considered European. All samples prior to 1994 appear to be European while all those after 1994, except one, seem to be Africanized. The gap in samples from 1980 to 1993 may be due to a reduction in honeybees caused by *Varroa* mites. Figure 1(B) The ratio of honeybees (gray) and native bees (black) in museum collections in three periods (1. European bees: 1900–1984; 2. *Varroa* present: 1985–1993; 3. Africanized bees present: 1994–2003). Native bees are collected at similar numbers across the years (see text). Before 1930, there were very few bee specimens in these collections. Proportion of *Apis mellifera* specimens to native bee specimens across the three periods were significantly different (likelihood ratio test χ^2^ = 259.28, df = 2, *P* < 0.0001 *N* = 609). Each of the three periods was significantly different from each other in *post hoc* comparisons.

After preliminary tests demonstrated lack of Western European mtDNA in samples (*N* = 15), the RFLP analysis of the mitochondrial DNA on all colonies (*N* = 112) sampled across Puerto Rico (Fig. 1) demonstrated them to be Africanized by maternal descent. This was expected because a complete replacement of European races by Africanized bees within 5 years has been observed in other places such as southern USA (Pinto et al. 2005; Whitfield et al. 2006). We did not expect to find large numbers of feral European colonies because *Varroa* mites eliminated most of them (Martin et al. 1998), and there were only ∼4000 managed colonies that could serve as sources of swarms, and a portion of these could have been European bees (Departamento de Agricultura de Puerto Rico 2006).

However, for the nuclear locus studied, we found 65.6% (*N* = 32 alleles sampled) to have the pattern typical of European bees; the others had only one of the five possible African-specific fragment types (the A, or 1050-bp fragment produced when the S-3 locus is cut with Alu1, as per Suazo and Hall 2002). This level of European alleles for a nuclear locus in an Africanized population is high compared to other studies (Pinto et al. 2005; Whitfield et al. 2006; Francoy et al. 2009).

### Wing size

Although we found high frequency of European alleles in the genomic locus examined, wing lengths considered a polygenic trait (Sylvester and Rinderer 1987) were typical of Africanized bees, and the measurements on current samples were not different from museum samples after 1994 the year when Africanized were reported to have arrived on the island. The smaller size of Africanized bees has been proposed to relate to their metabolism, an adaptation related to their tropical origins Harrison and Hall 1993). According to the FABIS method (Sylvester and Rinderer 1987), Africanized bees generally have shorter wings (<9 mm) when compared with European races (>9 mm). The average wing length of the Africanized bees we studied was 8.72 mm (SD = 0.33 mm; *N* = 244, 8–9 bees from each of 28 colonies).

Wings measured from specimens in museums collected before 1994 are similar in length to bees of European origin (≥9 mm). In contrast, the bees collected after 1994 have shorter wings typical of Africanized bees (<9 mm; except one sample from 1994; Fig. 1A). There was a paucity of honeybee specimens in museum collections from the period following the arrival of *Varroa* mites in Puerto Rico in 1984 (Martin et al. 1998) and the arrival of Africanized bees in 1994 (Cox 1994) (Fig. 1A,B). Only after reported arrival date of Africanized bees did, we find again honeybee specimens in museums at numbers similar to levels before 1984. The three intervals shown in Fig. 1(B), in order, correspond to 1 (1900–1983): European bees present, *Varroa* mites and Africanized bees absent; 2 (1984–1993): Decade when *Varroa* mites are present, Africanized bees absent; 3 (1994–2003): Decade when *Varroa* mites and Africanized bees are present. Statistically significant underrepresentation of honeybees is evident for interval 2 when *Varroa* was present and Africanized bees were not, and overrepresentation is detected in the last time interval. This pattern could not be explained by changes in collection effort over the years because in these same time intervals, there was no noticeable change in the numbers of specimens of native bee species in the collections examined (Fig. 1B, a distribution analysis of native bees collected in blocks of 10 years, from 2003 going back to 1930, indicates no significant differences in numbers over the years). The museum data corroborate the virtual complete Africanization of the honeybees on the island since 1994, based on independently collected bee samples across the island.

### Defensive behavior

**Figure 2:**
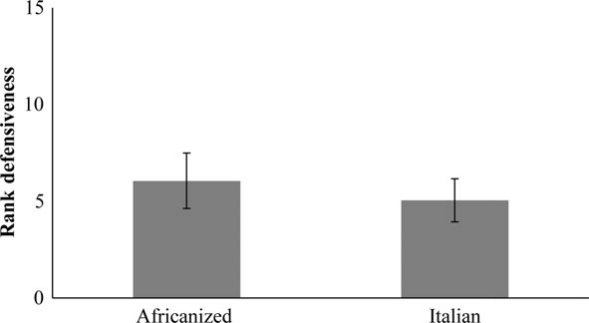
Average defensiveness rank of honeybees in Puerto Rico. Africanized bees (gray) and Italian bees (white) are compared. The average defensive rank of the Africanized bees is not significantly different to the average defensive rank of the Italian bees (statistics in the text).

We evaluated defensive behavior with a repeated behavioral rank assay and corroborated the results with a repeated sting assay (Giray et al. 2000). The behavioral assay is considered a more reliable measure than the sting assay because repeated measurements show lower variation than with sting assay or other assays examined (Guzmán-Novoa et al. 2003). The behavioral assay ranks four components of defensive behavior that reflect the main defensive behaviors (i.e. stinging and guarding) (Giray et al. 2000; Guzmán-Novoa et al. 2003). *A priori* power tests using the magnitude of differences observed across Africanized and European bees in the Mexican study, where the same assay had been used, showed that at sample sizes of only four colonies per race, the power of comparison was near one. We used 10 colonies of each race. The results of the rank assay suggest that the Africanized bees of Puerto Rico have reduced defense. The average defensive score of the Africanized bees (average = 6.06, *N* = 9, one outlier is excluded, including this score does not change statistical analysis results) was not significantly different from the average rank of the Italian bees in Puerto Rico (average = 5.05, *N* = 10) and the European bees of Mexico (average = 5.05; *N* = 12) yet was significantly lower than the Africanized bees of Mexico (Giray et al. 2000) (average = 11.75, *N* = 12; Wilcoxon signed-rank test, χ^2^ approximation = 23.66, df = 3, *P* > 0.0001; Fig. 2).

We corroborated the results of the behavioral rank assay with a sting assay which we compared with results from European bees in Illinois (Giray et al. 2000). There was no significant difference between the average number of stings of the Africanized bees of Puerto Rico (average = 220.4, SD = 141.7, *N* = 9) and European bees studied in Illinois (average = 197.1, SD = 201.4, *N* = 13; *t* = 0.273, *P* = 0.788). The time to sting was compared with a *t*-test (Zar 1999) to data from Giray et al. (2000); communicated by T. Giray). The average time to first sting of the Africanized bees in Puerto Rico was 56.6 s (SD = 44, *N* = 9). The time to sting was not different to European bees (mean 57.7 SD = 56.7 *N* = 10; see Table 1; *t* = 0.04, df = 17, *P* > 0.96). Average time to first sting results from this study, and from Illinois, European bees were not different from European bees studied by Guzmán-Novoa and Page (1993). This time to first sting for Puerto Rican Africanized bees (56.6 s) contrasts greatly with time to first sting data from other studies with Africanized bees (range: 1.53–11.3 s, see Table 1, studies denoted by an ‘a’).

In a comparison of effect size difference in defensiveness of Africanized and European bees, either for colonies compared in the same location or in different locations, and by different assays and sample sizes, we found that the average effect size in Puerto Rico study is the lowest, and an outlier in all comparisons of Africanized versus European bees (Table 1, Figure S2).

### *Varroa* mite infestation and resistance mechanisms

Mite resistance is considered a typical trait of Africanized bees (Guzmán-Novoa et al. 1999). We examined *Varroa* mite infestation of the brood (Mondragón et al. 2005) and adult worker bees (Martin and Medina 2004). Infestation was low in brood (0.7% prevalence in of sampled brood cells) and on workers (average abundance = 75.40 mites per colony, SD = 31.6 mites, *N* = 9). A high percentage of fallen mites collected over night had carapace or leg damage (74.7%), suggesting that the bees damaged the mites (Martin and Medina 2004).

**Figure 3:**
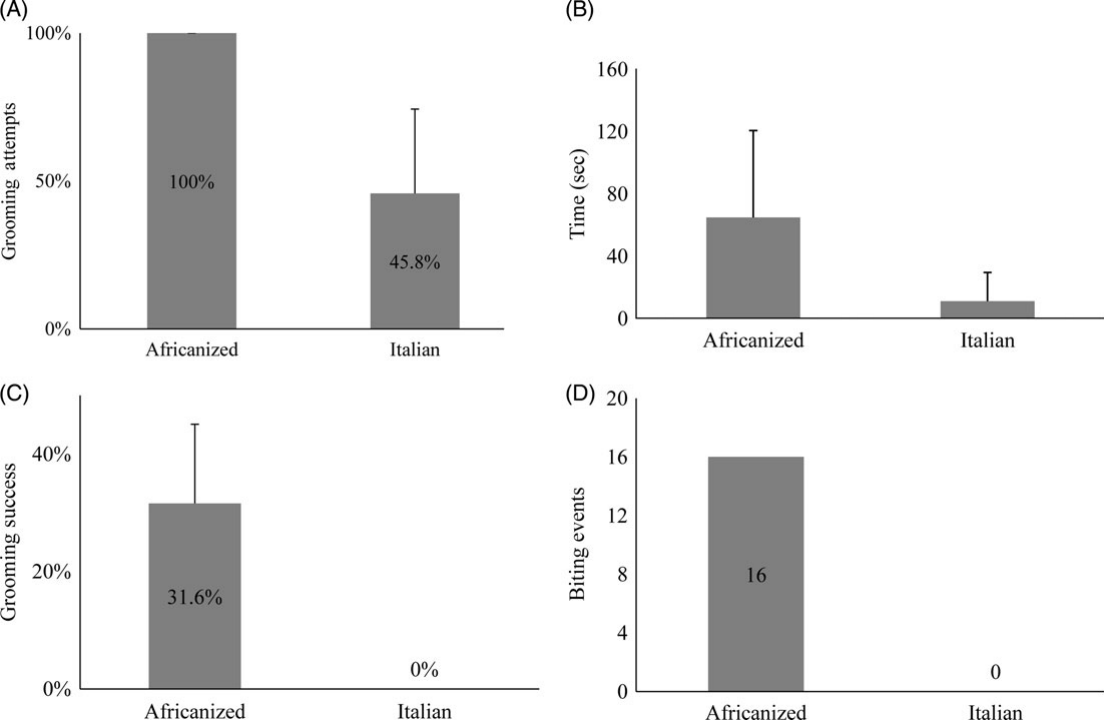
Grooming behavior of Africanized (gray) and Italian (white) bees. (A) Africanized bees attempted grooming more often, (B) spent significantly more time grooming (*N* = 57, *t* = 5.42, *P* < 0.001), (C) groomed with more success, and (D) demonstrated biting behavior.

After determining that mite infestation levels were low, we evaluated possible mechanisms of resistance including hygienic behavior (Spivak and Reuter 1998; Aumeier 2001) and grooming behavior (Martin and Medina 2004). The studied colonies demonstrated low levels of hygienic behavior (average brood removal within two days was only 57.44%, SD = 21.76%, *N* = 9 colonies). Although the colonies were not hygienic, grooming behavior appeared to be an important mechanism of resistance. In controlled assays comparing Africanized bees and Italian bees, we observed Africanized bees autogrooming *Varroa destructor* mites from their bodies (Movie S1), similar to more defensive Africanized bees (Mondragón et al. 2005). They also attacked and bit the mites without provocation (Movie S2). In every trial, the Africanized bees attempted autogrooming (*N* = 40) while the European bees attempted autogrooming in only 45.8% (SD = 28.5%, *N* = 38; Fig. 3A) of the trials. The time spent autogrooming was significantly longer for Africanized bees than Italian bees (Fig. 3B). By spending more time autogrooming, the Africanized bees should increase their probability of mite removal. Africanized bees groomed successfully in 31.6% (SD = 13.5%, *N* = 40; Fig. 3C) of the trials, whereas the European bees never successfully remove mites (Movie S3). Moreover, Africanized bees attacked and bit mites in 16 trials with bees of four different colonies (Fig. 3D); European bees were not observed performing this behavior. We did not, however, observe allo-grooming among the assayed bees. In a separate assay of Africanized bees, results suggest that neither autogrooming nor biting is associated with a particular age-related job. In three additional colonies assayed for mite biting behavior, the average frequency of grooming (anova: *F* = 0.7956, *P* = 0.4645) and the average biting events (anova: *F* = 0.7033, *P* = 0.5049) did not differ among nurses, guards, and foragers. Successful removal of ectoparasites by Africanized bees seems to be a general defense important for bees of different ages and jobs. Mite biting was performed by some individuals in all seven Africanized bee colonies tested in the grooming and biting comparisons under both white light and under red light.

## Discussion

We infer from the combined evidence that under island conditions of Puerto Rico, the typically linked traits of Africanized bees – defense, small size, and parasite resistance – have changed independently. Studies on evolutionary processes such as drift, founder effect, and selection could bring explanations for this unique phenomenon. The honeybees of the island of Puerto Rico are Africanized by maternal descent (i.e. mtDNA) yet with a fairly high frequency of European alleles at the studied nDNA locus. Their average wing length and resistance to mites are typical of Africanized bees. However, they have reduced defensive behavior in comparison with Africanized bees elsewhere.

Should the high levels of European alleles at the studied genomic locus be representative of other loci, this may be due to drift or a founder effect. Although unlikely, it is possible that the bees arrived with high levels of European alleles, similar to those found in the early 1990s in the recently arriving populations of Africanized honeybees to southern USA (Whitfield et al. 2006). Alternately, the arriving Africanized bees may have mated with the remnant European bees on the island. In support of the founder effect, although the sample size is small, only one of the five alleles of African origin at this locus has been recorded in our study.

The second or ‘mating on the island’ alternative would be similar to theoretical scenarios where one smaller population spreads and introgression of private alleles from the larger population occurs prior to spread (Forhan et al. 2008). The calculated introgression at this locus based on the European private allele is 65% (as per Slatkin and Barton 1989; see also Martinez et al. 2001).

Although at the locus related to defensive behavior, there is high European allelic introgression, the sampled bees have typical wing lengths of Africanized bees. Wing length is a polygenic trait and is highly correlated with body size. Body size in turn is related to metabolic capacity, where smaller bees tend to have higher metabolic capacities than larger ones (Harrison and Hall 1993). The higher metabolic capacity typically favored in the tropics should also be favored on islands where frequently selection for smaller-sized individuals occurs (McNab 1994). Alternately, albeit unlikely, it may be that by chance, European genes present in the population are not related to size.

Mites were present in the studied Africanized colonies, but the levels were low. Grooming and biting rather than hygienic behavior seem to be the mechanisms that convey resistance to *Varroa* mites. The studied Africanized bees autogroomed for longer bouts and with more success than European bees. The Africanized bees also bit and damaged mites. Moreover, because half the biting occurred before grooming, it is possible that the bees remove the mites from the colony; this could explain the low mite population levels observed in this and other studies (e.g. Mondragón et al. 2005) of Africanized bees. The difference across individuals in a colony for mite biting could be due to epigenetic (rev. for worker behavior in Hunt 2007) or genetic differences as with other behaviors (e.g. guarding and undertaking; Robinson and Page 1988), and rate of behavioral development (Giray et al. 1999, 2000). Mite resistance, considered a favorable trait of Africanized honeybees, seems to be favored on the island where these mites are prevalent. Mites could be an important selective agent also in European bees; some beekeepers began to eliminate or reduce acaricide use, and a few feral European colonies were found to survive even in areas with mites (De Jong and Soares 1997). In these cases, colony survival may be due to resistance from the bees (De Jong and Soares 1997) or changes in the mites (c.f. Seeley 2006). Nevertheless, extensive studies on other traits of *Varroa*-resistant colonies or populations of honeybees are lacking (c.f. Le Conte et al. 2007).

The reduced defensive behavior of Africanized bees in Puerto Rico may be due to drift where the African alleles may have been diluted through hybridization with European bees. However, the maintenance of the other typical traits (i.e. wing size and mite resistance) seems to contradict drift as the only mechanism affecting genetic traits in the population. The retention of these traits and the conditions related to living on an oceanic island suggest that selection may favor the gentle behavior of these bees. Defense in organisms is important for survival against natural enemies. Defense, however, is energetically costly (rev. by Rivera-Marchand et al. 2008) particularly on islands where resources can be limited. In the honeybee colony, older individuals take on higher risks including colony defense and foraging for food (Winston 1987). Because individuals that defend the colony can potentially forage, highly defensive colonies tend to invest less in foraging than gentler colonies (Rivera-Marchand et al. 2008). As a result, allocation of workers to defense or foraging may represent a trade-off (Giray et al. 2000; Rivera-Marchand et al. 2008) that is especially important under island conditions. Although the amount of time for selection to potentially affect the Africanized bees on Puerto Rico seems short, rapid evolution (i.e. changes that adapt organisms within a few generations to changing or new environments) has been reported for organisms extending from simple to complex, plant to animal (rev. in Hairston et al. 2005; see Jones et al. 2001).

In conclusion, we demonstrate that in the oceanic island of Puerto Rico, defense and other tropical adaptations observed in other Africanized bee populations are uncoupled. This could be due to a genetic drift, founder effect, or strong selection for foraging and for ectoparasite resistance (De Guzman et al. 1997; Anderson and Trueman 2000). The recently completed genome project (Honey Bee Genome Sequence Consortium 2006) and a genome-wide analysis of variation in this population and mainland populations (see Whitfield et al. 2006; Hohenlohe et al. 2010) could help resolve the contribution of different processes to the mosaic of traits observed in Puerto Rican honeybees.

Studies on Africanized bees on this (and other) oceanic island(s) provide a new model for the study of behavioral evolution on islands. Understanding these gentle bees that can maintain low populations of mites thereby escaping both chemical treatments and possibly viral infections is of topical interest. This is especially important because of recent concerns that many factors including *Varroa* mites, viral diseases (transmitted by mites), and chemicals used to control mites negatively affect bee health (Giray et al. 2010; Neumann and Carreck 2010 and references there in Bromenshenk et al. 2010; Mullin et al. 2010; Aizen and Harder 2009).

## Data archiving statement

Data deposited in the Dryad repository: doi:10.5061/dryad.d6q7q72g.

## Supporting Information

**Figure S1.** Honeybee sampling sites. Africanized honeybees on the island of Puerto Rico were collected from 112 feral hives (shaded municipalities). Puerto Rico is in the Caribbean Sea (see the inset), 1662 km South of Florida,127 km East of Dominican Republic. The length is 177 km and the width is 64 km. To ensure colony origin, we only sampled pollen foragers returning to or young bees inside the hive. The two islands to the East, Culebra and Vieques were also sampled.

**Figure S2.** Distribution of log transformed (average) effect sizes in comparisons of Africanized and European bees in different studies (see Table 1). The average effect size from the current study is an outlier (identified with a black square in the box quartile plot above the histogram, Grubb’s test: *N* = 9, Mean log*d* = 1.055 SD = 0.92, for Puerto Rico log *d* = −1.022, *z* = 2.253 *P* < 0.05). The box quartile plot indicates±95% confidence interval for the mean with tips of the diamond. The box and whiskers show ±1 SD. The effect size data were log transformed for normality and analyzed with the Shapiro-Wilk test (*W* = 0.88,*P* = 0.14).

**Movie S1.** Grooming behavior of Africanized honeybees. An Africanized bee is seen successfully grooming off a Varroa mite.

**Movie S2.** Biting behavior of Africanized honeybees. An Africanized bee is seen biting a Varroa mite.

**Movie S3.** Grooming behavior of Italian honeybees. A Varroa mite is seen attaching itself to an Italian bee that does not attempt to groom it from its body.
