# Supplementary figures and images for: Gentle Africanized bees on an oceanic island
Source: Evol Appl. 2012 Nov;5(7):746–56. doi: 10.1111/j.1752-4571.2012.00252.x (PMC3492899; doi:10.1111/j.1752-4571.2012.00252.x)

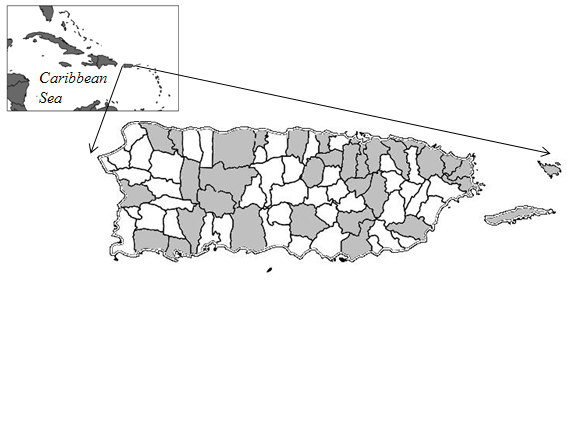

Supplement: Supplementary file 1 [file eva0005-0746-SD1.tif]

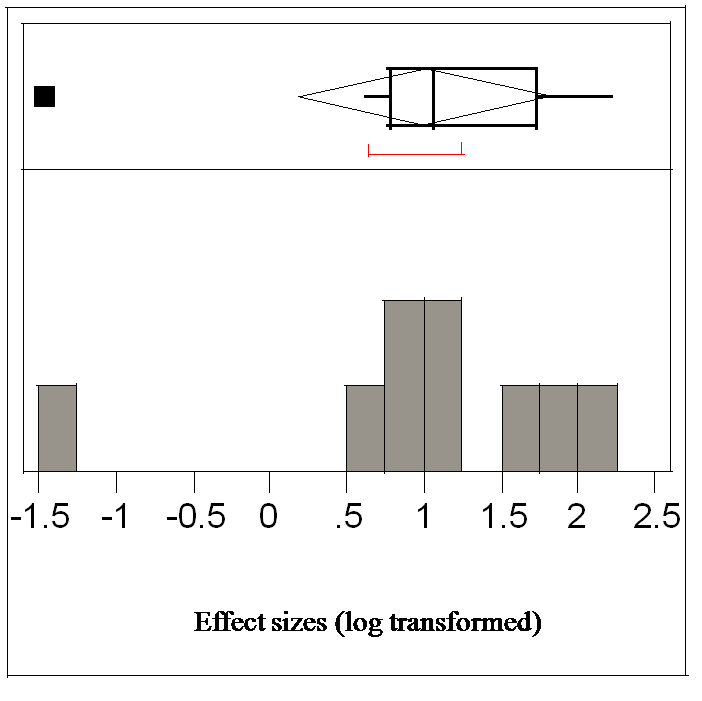

Supplement: Supplementary file 2 [file eva0005-0746-SD2.tif]
